# AAV-mediated YAP expression in cardiac fibroblasts promotes inflammation and increases fibrosis

**DOI:** 10.1038/s41598-021-89989-5

**Published:** 2021-05-18

**Authors:** Jamie Francisco, Yu Zhang, Yasuki Nakada, Jae Im Jeong, Chun-Yang Huang, Andreas Ivessa, Shinichi Oka, Gopal J. Babu, Dominic P. Del Re

**Affiliations:** Department of Cell Biology and Molecular Medicine, Cardiovascular Research Institute, Rutgers New Jersey Medical School, 185 South Orange Avenue, MSB G-609, Newark, NJ 07103 USA

**Keywords:** Cell biology, Cardiology, Cardiovascular diseases

## Abstract

Fibrosis is a hallmark of heart disease independent of etiology and is thought to contribute to impaired cardiac dysfunction and development of heart failure. However, the underlying mechanisms that regulate the differentiation of fibroblasts to myofibroblasts and fibrotic responses remain incompletely defined. As a result, effective treatments to mitigate excessive fibrosis are lacking. We recently demonstrated that the Hippo pathway effector Yes-associated protein (YAP) is an important mediator of myofibroblast differentiation and fibrosis in the infarcted heart. Yet, whether YAP activation in cardiac fibroblasts is sufficient to drive fibrosis, and how fibroblast YAP affects myocardial inflammation, a significant component of adverse cardiac remodeling, are largely unknown. In this study, we leveraged adeno-associated virus (AAV) to target cardiac fibroblasts and demonstrate that chronic YAP expression upregulated indices of fibrosis and inflammation in the absence of additional stress. YAP occupied the *Ccl2* gene and promoted Ccl2 expression, which was associated with increased macrophage infiltration, pro-inflammatory cytokine expression, collagen deposition, and cardiac dysfunction in mice with cardiac fibroblast-targeted YAP overexpression. These results are consistent with other recent reports and extend our understanding of YAP function in modulating fibrotic and inflammatory responses in the heart.

## Introduction

Fibrosis is associated with heart disease independent of etiology and is recognized as a fundamental driver of cardiac pathology^1^. It is a process that results from fibroblast to myofibroblast transition, enhanced myofibroblast activity, and the aberrant deposition of extracellular matrix (ECM) proteins^2^. Excessive fibrosis can impair heart contractility and relaxation and facilitate the progression to failure. Conversely, fibrosis is necessary for wound healing after cardiac insult, e.g. to replace the massive loss of cardiomyocytes following myocardial infarction (MI), and an insufficient response can lead to heart wall rupture and death^3^. Therefore, the ability to modulate the extent and duration of the fibrotic response could prove advantageous in mitigating cardiac dysfunction. Limitations in our understanding of the cellular and molecular underpinnings of myocardial fibrosis continue to preclude effective therapeutic options for clinical use.

There is extensive literature investigating molecular mechanisms that regulate myofibroblast differentiation and fibrosis in multiple organs including the heart. In the context of myocardial fibrosis, studies have highlighted the importance of several pathways critical for myofibroblast activation including calcium-dependent signaling^4–6^, canonical and non-canonical TGFβ signaling^7–9^, and mechanotransduction-related mechanisms^10–13^. Importantly, fibrosis is also associated with tissue inflammation, and there is thought to be coordinated and complex crosstalk that occurs between fibroblasts/myofibroblasts and immune cells to modulate inflammation and fibrosis in cardiac injury, remodeling, and failure^14^.

Recent work including our own has demonstrated the importance of Yes-associated protein (YAP)-TEA domain transcription factor (TEAD) signaling in the context of cardiac fibrosis and myofibroblast differentiation^12,15^. We reported that YAP is activated in cardiac fibroblasts in response to MI or neuroendocrine stimulation and that inhibition of YAP in this compartment attenuates fibrosis and dysfunction. However, whether YAP activation is sufficient to drive fibrosis, and whether YAP-induced myofibroblast differentiation influences inflammation within the myocardium, remain largely unaddressed. The main objectives of the current study were to investigate the feasibility of cardiac fibroblast selective expression of YAP using an AAV-based approach and to determine whether YAP activation in cardiac fibroblasts in the absence of additional stress was sufficient to modulate cardiac fibrosis and inflammation. To our knowledge, this is the first study to examine the effect of increased cardiac fibroblast YAP expression in vivo.

## Methods

### Adeno-associated virus

The pAAV-hTCF21 promoter vector was generated by cloning the 1013 bp human TCF21 (hTCF21) promoter fragment (Switchgear Genomics, Cat# S712020) into the ClaI and EcoR1 sites of the pAAV-MCS-Promoterless vector (Cell Biolabs, Cat# VPK-411). The FLAG-YAP(S127A) cDNA (Addgene 27370) was cloned to the EcoRI and BamHI sites of the pAAV-hTCF21 promoter vector. The GFP cDNA was cloned into the SalI and Hind III sites of the pAAV-hTCF21 promoter vector. Recombinant AAV was produced using the AAV-DJ/8 helper-free expression system (Cell Biolabs, Inc.) as described^16^. Briefly, the recombinant pAAV-hTCF21 vector was co-transfected into 293AAV cell line along with pAAV-DJ/8 and pHelper in a 1:1:1 ratio using polyethylenimine. After 72 h of transfection, cells were harvested and purified by the iodixanol gradient/ultra-centrifugation method. The AAV fraction obtained from the gradient centrifugation was concentrated by the Vivaspin 20 concentrator (100 kDa cut-off, Sartorius, Germany). The virus titer was determined using the Cell Biolabs AAV quantitation kit (Cat. # VPK-145).

### Animals

Wild type C57BL/6J male mice were purchased from Jackson Labs (stock: 000664). At 4 months of age, mice were anesthetized using 12 μL/g body weight of 2.5% tribromoethanol (Avertin, Sigma), and administered AAV-hTCF21-GFP or AAV-hTCF21-FLAG-YAP(S127A) retro-orbital at a dose of 1 × 10^11^ vg/mouse. All mice were age-matched. Due to limited resources, only male mice were used for these experiments, as we have done previously for YAP cardiac studies^17–20^. Mice were housed in a temperature-controlled environment with 12-h light/dark cycles where they received food and water ad libitum. All protocols concerning the use of animals were approved by the Institutional Animal Care and Use Committee at Rutgers, The State University of New Jersey. All animal experiments were performed in accordance with approved protocols and complied with the ARRIVE guidelines.

### Echocardiography

Mice were anesthetized using 2.5% Avertin (0.012 mL/g body weight; i.p.), and echocardiography was performed as described previously^21^. Two-dimensional guided M-mode measurements of LV internal diameter were obtained from at least three beats and then averaged. LV end-diastolic dimension (LVEDD) was measured at the time of the apparent maximal LV diastolic dimension, and LV end-systolic dimension (LVESD) was measured at the time of the most anterior systolic excursion of the posterior wall. LV ejection fraction (LVEF) was calculated using the following formula: *LVEF* (%) = 100 × (*LVEDD*^3^ − *LVESD*^3^)/*LVEDD*^3^.

### Adult mouse heart cell isolation

The isolation and culture of adult mouse cardiomyocytes and cardiac non-myocytes were conducted according to the protocol described in Ackers-Johnson et al.^22^. This method utilizes direct needle perfusion of the left ventricle ex vivo, which allows for the isolation, separation, and culture of adult mouse cardiomyocytes and non-myocytes. Following digestion, atria and right ventricle were removed, and the left ventricle isolated. The cell suspension was filtered through a 100 μm strainer, and cardiomyocytes were separated from non-myocytes by repeated rounds of gravity settling. The purity of cellular fractions was determined by mRNA expression (*Tnnt2*) and protein expression (cardiac troponin T, PDGFRα and Hsp47). Both cardiomyocyte and non-myocyte fractions were used for immunoblotting and qPCR analysis.

### Cell culture and reagents

Primary cultures of ventricular cardiac fibroblasts were prepared from 1-day-old (WI)BR-Wistar rats and maintained in culture as described previously^20^. Cell passages 2 or 3 were used for experiments. For YAP knockdown experiments, fibroblasts were transduced with control scramble (shCT) short hairpin RNA (shRNA) or YAP shRNA. After 24 h, cells were treated with TGFβ1 (10 ng/mL, R&D Systems) or vehicle control for an additional 24 h, and then collected for RNA isolation. For YAP overexpression experiments, cells were transduced with YAP(S127A) or control LacZ adenovirus as described previously^18^.

### Histological analyses

Heart and liver specimens were fixed with formalin, embedded in paraffin, and sectioned at 6 µm thickness. Hematoxylin and eosin (H&E) staining was used to visualize the myocardial structure. Interstitial fibrosis was evaluated by picrosirius red or Masson’s Trichrome staining as described^23^.

### Immunostaining

Mouse hearts were fixed in formalin and sectioned at 6 μm thickness. Tissue sections were then subjected to deparaffinization and antigen unmasking using citrate buffer and washed with PBS containing 0.3% Triton X-100. Immunostaining in cardiac fibroblasts and cardiomyocytes was performed as described previously^15^. Briefly, cells were fixed in paraformaldehyde and permeabilized using PBS containing 0.3% Triton X-100. Samples were blocked with 5% BSA and incubated with primary antibody overnight and with Alexa Fluor 488- or Alexa Fluor 568-conjugated secondary antibodies (Molecular Probes) at room temperature the following day. Primary antibodies used were rabbit monoclonal anti-FLAG antibody (D6W5B, Cell Signaling #14793), rabbit monoclonal anti-GFP antibody (Cell Signaling #2956), rat monoclonal anti-CD68 antibody (Biolegend #137001), mouse monoclonal anti-Hsp47 antibody (M16.10A1, Enzo Life Sciences #ADI-SPA-470), mouse monoclonal anti-Troponin-T antibody (13-11, Thermo Fisher MA5-12960), mouse monoclonal anti-αSMA antibody (clone 1A4, Sigma), mouse monoclonal anti-FLAG antibody (Sigma, F1804), rabbit polyclonal anti-CD31 antibody (Abcam, ab28364), rabbit monoclonal anti-Ki67 antibody (Cell Signaling #9129), and rabbit polyclonal anti-Troponin-I antibody (Santa Cruz, sc-15368). Nuclei were stained with DAPI. Imaging was performed using a Nikon Eclipse Ti fluorescence microscope and NIS-Elements imaging software.

### Immunoblotting

Tissue or cells were homogenized in lysis buffer containing 50 mmol/L Tris–HCl (pH 7.5), 150 mmol/L NaCl, 1% IGEPAL CA-630, 0.1% SDS, 0.5% deoxycholic acid, 1 mmol/L EDTA, 0.1 mmol/L Na_3_VO_4_, 1 mmol/L NaF, 50 μmol/L phenylmethylsulfonyl fluoride (PMSF), 5 μg/mL aprotinin, and 5 μg/mL leupeptin. Following SDS-PAGE, immunoblotting was performed using the following antibodies: anti-YAP (Cell Signaling #14074, 1:1000), anti-cardiac troponin T (Thermo Fisher MA5-12960, 1:2000), anti-GFP (Cell Signaling #2956, 1:1000), anti-GAPDH (Cell Signaling #5174, 1:1000), anti-PDGFRα (Cell Signaling #3174, 1:1000), anti-Hsp47 (Enzo Life Sciences #ADI-SPA-470, 1:1000), and anti-tubulin (Sigma, T-6199, 1:1000). Densitometry was performed using NIH ImageJ software.

### qRT-PCR

RNA was isolated from tissue or cells using TRIzol (Thermo Fisher). Concentration and purity of RNA were determined (NanoDrop 2000, Thermo Fisher) and cDNA was generated. Quantitative real-time PCR was performed as described previously^20^. Targets were normalized to internal control (*18s*), and relative quantitation was determined using the comparative ΔΔC_T_ method. The following primers were used:

*Il-1b* (rat): 5′-TGCAGGCTTCGAGATGAAC-3′; 5′-GGGATTTTGTCGTTGCTTGTC-3′.

*Tnfa* (rat): 5′-CTTCTCATTCCTGCTCGTGG-3′; 5′-TGATCTGAGTGTGAGGGTCTG-3′.

*Il-6* (rat): 5′-AAGCCAGAGTCATTCAGAGC-3′; 5′-GTCCTTAGCCACTCCTTCTG-3′.

*Ccl2* (rat): 5′-ATGCAGTTAATGCCCCACTC-3′; 5′-TTCCTTATTGGGGTCAGCAC-3′.

*Ccl5* (rat): 5′-ATATGGCTCGGACACCACTC-3′; 5′-CCACTTCTTCTCTGGGTTGG-3′.

*Cxcl2* (rat): 5′-AGGGTACAGGGGTTGTTGTG-3′; 5′-TTTGGACGATCCTCTGAACC-3′.

*18s* (rat): 5′-CATTCGAACGTCTGCCCTAT-3′; 5′-GTTTCTCAGGCTCCCTCTCC-3′.

*Col1a1* (ms): 5′-GCTCCTCTTAGGGGCCACT-3′; 5′-CCACGTCTCACCATTGGGG-3′.

*Col3a1* (ms): 5′-CTGTAACATGGAAACTGGGGAAA-3′; 5′-CCATAGCTGAACTGAAAACCACC-3′.

*Tgfβ1* (ms): 5′-CTCCCGTGGCTTCTAGTGC-3′; 5′-GCCTTAGTTTGGACAGGATCTG-3′.

*Fn1* (ms): 5′-ATGTGGACCCCTCCTGATAGT-3′; 5′-GCCCAGTGATTTCAGCAAAGG-3′.

*Ccl2* (ms): 5′-CATCCACGTGTTGGCTCA-3′; 5′-AACTACAGCTTCTTTGGGACA-3′.

*Ccl5* (ms): 5′-GCTGCTTTGCCTACCTCTCC-3′; 5′-TCGAGTGACAAACACGACTGC-3′.

*Cxcl2* (ms): 5′-CTTTCCAGGTCAGTTAGCCTT-3′; 5′-CAGAAGTCATAGCCACTCTCAAG-3′.

*Il-1b* (ms): 5′-GACCTGTTCTTTGAAGTTGACG-3′; 5′-CTCTTGTTGATGTGCTGCTG-3′.

*Yap* (ms): 5′-TACTGATGCAGGTACTGCGG-3′; 5′-TCAGGGATCTCAAAGGAGGAC-3′.

*Tnnt2* (ms): 5′-CAGAGGAGGCCAACGTAGAAG-3′; 5′-CTCCATCGGGGATCTTGGGT-3′.

*18s* (ms): 5′-GTAACCCGTTGAACCCCATT-3′; 5′-CCATCCAATCGGTAGTAGCG-3′.

*FLAG-Yap*: 5′-GGACTACAAAGACGATGAC-3′; 5′-GCGGACGTGCACGATCTGAT-3′.

*GFP*: 5′-AAGCTGACCCTGAAGTTCATCTGC-3′; 5′-CTTGTAGTTGCCGTCGTCCTTGAA-3′

### Chromatin immunoprecipitation assay

ChIP assay was performed using the SimpleChIP Plus Enzymatic Chromatin IP Kit (Cell Signaling) according to the manufacturer’s instructions^15^. Briefly, neonatal rat cardiac fibroblasts were transduced with 3xFLAG-YAP(S127A) adenovirus or control LacZ for 24 h and then cells were cross-linked using 1% formaldehyde for 10 min. Cells were washed with 1 × PBS, and glycine was added to stop the cross-linking reaction. Cells were then scraped, nuclei were isolated and lysed, and sheared chromatin was isolated after sonication. Immunoprecipitation reactions were carried out using chromatin extracts and anti-YAP (Cell Signaling #14074) or control rabbit IgG (Cell Signaling #2729) antibodies overnight at 4 °C. The following primers were used to perform quantitative PCR: 5′-AGACATTCCAGATGGCTCTCTCCT-3′ and 5′-AAACATTCCTTTTGATTTGGTCACGATGAAC-3′. Results are presented as fold change relative to control (LacZ).

### Luciferase reporter assay

HEK293 cells (ATCC CRL-3216) were transfected using Lipofectamine2000 reagent (Invitrogen) according to the manufacturer’s instructions. The 1.5 kb sequence immediately proximal to the first exon of the rat *Ccl2* gene was cloned into the XhoI and HindIII sites of the pGL3 Basic luciferase reporter plasmid (Promega). Cells were co-transfected with pCMV-eGFP (Addgene #13031) or pCMV-YAP(S127A) (Addgene #27370), and treated with verteporfin (Sigma #SML0534) or DMSO vehicle for 24 h. Following transfection and treatments, cells were lysed with Passive Lysis Buffer (Promega), and luciferase activity was measured using the luciferase assay system (Promega) with an OPTOCOMP I luminometer (MGM Instruments). The luminescence was normalized by total protein content.

### Statistics

All data are reported as mean ± standard error of the mean (SEM). Evaluation between three or more groups was done using one-way analysis of variance (ANOVA). Post-hoc multiple pairwise comparisons were performed using Tukey’s test. The student’s t-test was used to evaluate the difference in means between two groups. The normality of continuous variables was determined using the Shapiro–Wilk test. Statistical analyses were performed using Graph Pad Prism 8. A p-value < 0.05 was considered statistically significant.

## Results

### AAV-hTCF21-FLAG-YAP demonstrated selective expression in cardiac fibroblasts

**Figure 1:**
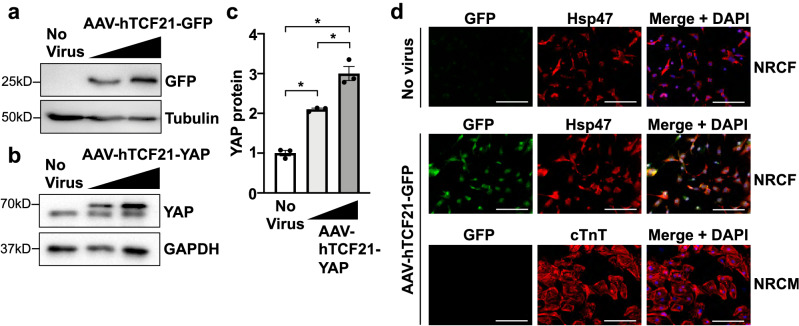
Characterization of AAV-hTCF21-GFP and AAV-hTCF21-FLAG-YAP expression in vitro. (**a–c**) Primary neonatal rat cardiac fibroblasts (NRCFs) were transduced with AAV-hTCF21-GFP or AAV-hTCF21-FLAG-YAP at 1 × 10^9^ or 3 × 10^9^ vg. The untreated (No virus) cells were used as controls. GFP or YAP was detected by western blot 24 h after virus addition. (**c**) Total YAP expression was quantified by densitometry. (**d**) NRCFs or neonatal rat cardiomyocytes (NRCMs) were transduced with AAV-hTCF21-GFP or untreated controls. GFP, Hsp47, or cardiac troponin T (cTnT) was visualized by fluorescence microscopy 3 days after virus addition. Scale bar, 100 μm. N = 3 experimental replicates. *p < 0.05. Full-length blots are presented in Supplementary Fig. S3.

The transcription factor, Tcf21, is essential for cardiac fibroblast cell fate^24^. Tcf21 is expressed in epicardial progenitor cells that give rise to cardiac fibroblasts, and its expression is maintained in mature fibroblasts^25^. Moreover, Tcf21 expression was shown to be significantly elevated in resident fibroblasts during the fibrotic phase^26^. Therefore, we utilized the human TCF21 promoter to generate a cardiac fibroblast-selective AAV. We first tested the expression of the AAV-hTCF21-GFP and the AAV-hTCF21-FLAG-YAP virus in cultured neonatal rat cardiac fibroblasts (NRCF) and neonatal rat cardiomyocytes (NRCM). After 24 h of AAV infection, we were able to detect GFP or FLAG-YAP expression by western blotting in the NRCF cultures (Fig. 1a–c). GFP was also visualized by fluorescence microscopy in AAV infected NRCFs, and colocalized with the fibroblast-selective marker heat shock protein 47 (Hsp47)^27^; however, we did not detect a considerable amount of GFP expression in the cardiomyocyte cultures indicating cell type selectivity of the hTCF21 promoter used in our AAV construct (Fig. 1d).

**Figure 2:**
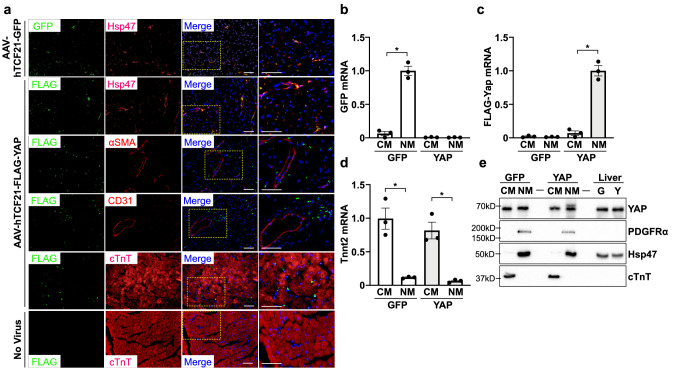
Characterization of AAV-hTCF21-GFP and AAV-hTCF21-FLAG-YAP expression in mouse hearts. Wild type C57BL/6J mice were administered AAV-hTCF21-GFP, AAV-hTCF21-FLAG-YAP, or no virus control. Hearts were excised and processed for immunostaining or cell isolation and fractionation. (**a**) Heart sections were stained to detect GFP or FLAG (green), and counterstained to visualize Hsp47, αSMA, CD31, or cardiac troponin T (red) and nuclei (DAPI). Right column panels are higher magnification merged images. Scale bar, 100 μm. (**b–e**) Heart cells were isolated from mice administered AAV-hTCF21-GFP or AAV-hTCF21-FLAG-YAP and separated into cardiomyocyte-enriched (CM) and non-myocyte-enriched (NM) fractions. RNA and protein were isolated for qPCR analysis and immunoblotting, respectively. (**b–d**) Expression of GFP, FLAG-Yap, and cardiac troponin T (Tnnt2) mRNA was determined by qPCR. (**e**) Protein fractions (20 μg per sample) were analyzed by western blot to detect YAP, PDGFRα, Hsp47, and cTnT protein levels. Whole liver homogenates from AAV-hTCF21-GFP and AAV-hTCF21-FLAG-YAP transduced mice (G and Y, respectively) were also analyzed (5 μg per sample). N = 3 mice/group. Representative blots are shown. *p < 0.05. Full-length blots are presented in Supplementary Fig. S3.

We next tested the expression of our constructs in vivo. AAV-hTCF21-FLAG-YAP or control AAV-hTCF21-GFP was administered to 4-month-old C57BL/6J wild type male mice via retro-orbital injection. To determine whether expression was selective, we first examined GFP or FLAG expression by immunofluorescence in heart sections of AAV-hTCF21-GFP treated, AAV-hTCF21-FLAG-YAP treated, or uninfected control mice. We could identify GFP-positive and FLAG-positive interstitial cells that displayed morphology consistent with cardiac fibroblasts in the myocardium of AAV-hTCF21-GFP and AAV-hTCF21-FLAG-YAP transduced mice, respectively, but did not detect FLAG-positive cells in the uninfected mice (Fig. 2a). The GFP or FLAG signal colocalized with the fibroblast selective marker Hsp47 but did not show appreciable overlap with the smooth muscle-selective marker αSMA, the endothelial selective marker CD31, or the cardiomyocyte-selective marker cardiac troponin T (cTnT), suggesting that the hTCF21-driven gene expression was selective for fibroblasts in vivo. We performed additional experiments that separated hearts from AAV-hTCF21-GFP and AAV-hTCF21-FLAG-YAP infected mice into cardiomyocyte-enriched and non-myocyte-enriched cell fractions. We used these fractions to detect GFP or FLAG-YAP mRNA by quantitative PCR. Our results demonstrate a significant enrichment of GFP mRNA in the AAV-hTCF21-GFP non-myocyte fraction, and FLAG-YAP mRNA in the AAV-hTCF21-FLAG-YAP non-myocyte-enriched fraction compared to the cardiomyocyte-enriched fractions (Fig. 2b,c). The purity of our cell separations was verified by cardiac troponin T (Tnnt2) mRNA detection (Fig. 2d). Protein from these cell fractions was also subjected to western blot analysis to determine FLAG-YAP expression. We found FLAG-YAP protein expression was restricted to the AAV-hTCF21-FLAG-YAP non-myocyte fraction, while no difference between cardiomyocyte-enriched fractions of each group was observed (Fig. 2e). Two markers of fibroblast-enrichment, Hsp47 and PDGFRα, along with cardiac troponin T, demonstrated fraction purity and equal protein loading. Taken together, these results indicate selective expression of FLAG-YAP in the fibroblast-enriched cardiac cell fraction.

### Increased expression of YAP in cardiac fibroblasts impaired heart function

To determine the possible impact of increased fibroblast YAP expression on cardiac function, echocardiography was performed at 4, 8 and 12 weeks after AAV administration. We found that AAV-hTCF21-FLAG-YAP transduced mice showed decreased LV ejection fraction relative to AAV-hTCF21-GFP treated mice, suggesting reduced systolic function (Supplementary Tables S1–S3). We also performed post-mortem analysis of these mice. We found no significant difference in heart weight, left ventricle weight, liver weight, or lung weight between AAV-hTCF21-FLAG-YAP and AAV-hTCF21-GFP treated mice (Supplementary Fig. S1a–d). Staining with H&E indicated no gross abnormalities or obvious differences in the integrity of the myocardium between treatment groups (Supplementary Fig. S1e).

### AAV-hTCF21-FLAG-YAP increased fibrosis and related gene expression in the heart

**Figure 3:**
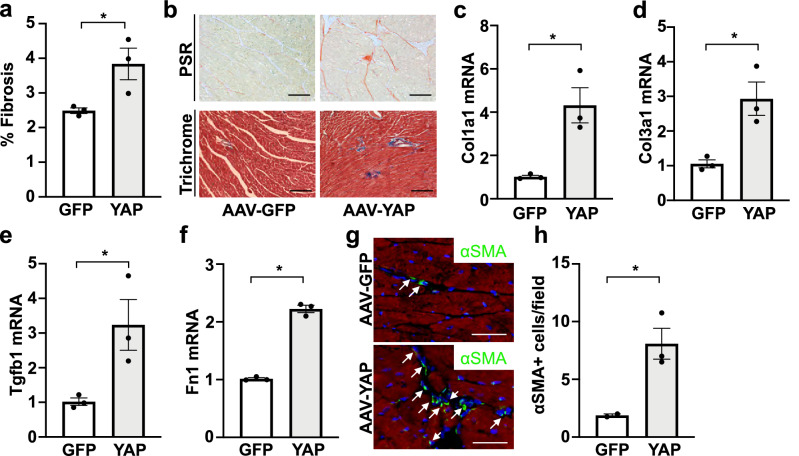
Indicators of fibrosis are upregulated in AAV-hTCF21-FLAG-YAP transduced hearts. Wild-type C57BL/6J mice were administered AAV-hTCF21-GFP or AAV-hTCF21-FLAG-YAP. (**a**,**b**) Hearts were stained with picrosirius red or Masson’s Trichrome to visualize collagen deposition. (**a**) The percent of picrosirius red (PSR)-positive staining was determined. (**b**) Representative images are shown. Scale bar, 200 μm. (**c–f**) RNA was isolated from LV tissue for qPCR analysis. (**g**,**h**) Hearts were sectioned for immunostaining to detect αSMA (green), cardiac troponin I (red), and nuclei (DAPI). White arrows identify αSMA-positive cells. Quantification of αSMA-positive cells in panel (**h**). Scale bar, 100 μm. N = 3 mice/group. *p < 0.05.

We next investigated whether increased fibroblast YAP expression could modify cardiac collagen deposition. Hearts from AAV-hTCF21-GFP and AAV-hTCF21-FLAG-YAP mice were stained with picrosirius red and the extent of fibrosis was quantified. We observed a significant increase in fibrosis in YAP expressing hearts compared to GFP control. Similar results were obtained using Masson’s Trichrome (Fig. 3a,b). We also measured the mRNA expression of fibrosis-associated genes. Our data demonstrate that Col1a1, Col3a1, TGFβ1 and fibronectin (Fn1) mRNA levels were significantly increased in YAP transduced hearts compared to control GFP hearts (Fig. 3c–f). In addition, we assessed the number of α smooth muscle actin (αSMA) positive cells within the myocardium, an indicator of myofibroblast differentiation. We observed a significant increase in αSMA positive cells in YAP expressing hearts compared to GFP controls (Fig. 3g,h). A similar analysis was performed in the livers of these mice. GFP or FLAG-YAP mRNA was detected in livers of AAV-hTCF21-GFP and AAV-hTCF21-FLAG-YAP treated mice, respectively; however, their levels were 80–90% lower than what was measured in corresponding heart tissue (Supplementary Fig. S2a,b). Moreover, endogenous Yap mRNA and protein amounts were comparable in livers from AAV-hTCF21-GFP and AAV-hTCF21-FLAG-YAP treated mice, and we were unable to detect FLAG-YAP protein expression in livers of AAV-hTCF21-FLAG-YAP treated mice (Supplementary Fig. S2c–e). Consistently, we did not observe any difference in liver collagen deposition or fibrotic gene expression between AAV-hTCF21-GFP and AAV-hTCF21-FLAG-YAP treated mice (Supplementary Fig. S2f–j). These results indicate that chronic cardiac fibroblast YAP expression can induce a fibrotic response in the myocardium in the absence of additional injury and that AAV-hTCF21-FLAG-YAP administration does not elicit this response in the mouse liver.

### YAP activation in cultured cardiac fibroblasts increased pro-fibrotic and pro-inflammatory gene expression

**Figure 4:**
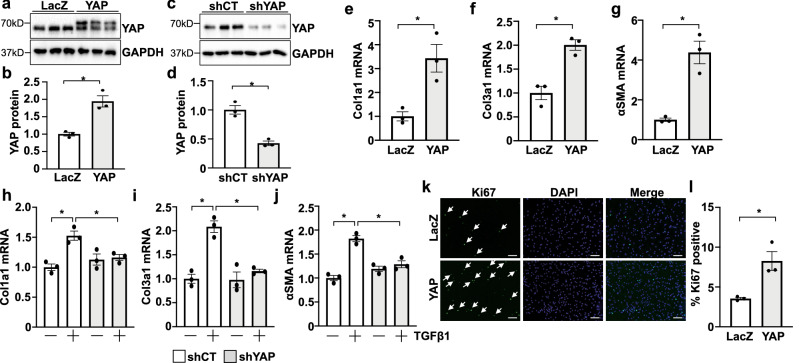
YAP mediates pro-fibrotic gene expression in cardiac fibroblasts. (**a–d**) Primary neonatal rat cardiac fibroblasts (NRCF) were subjected to adenoviral-mediated YAP overexpression or knockdown. Representative blots and quantification are shown. (**e–g**) YAP increased fibrosis-associated gene expression compared to LacZ control. (**h–j**) Depletion of endogenous YAP attenuated TGFβ1-induced expression of fibrosis-related genes. (**k**,**l**) YAP expression in NRCFs increased Ki67-positive nuclei compared to LacZ. White arrows indicate Ki67-positive nuclei. Scale bar, 100 μm. N = 3 experimental replicates. *p < 0.05. Full-length blots are presented in Supplementary Fig. S3.

**Figure 5:**
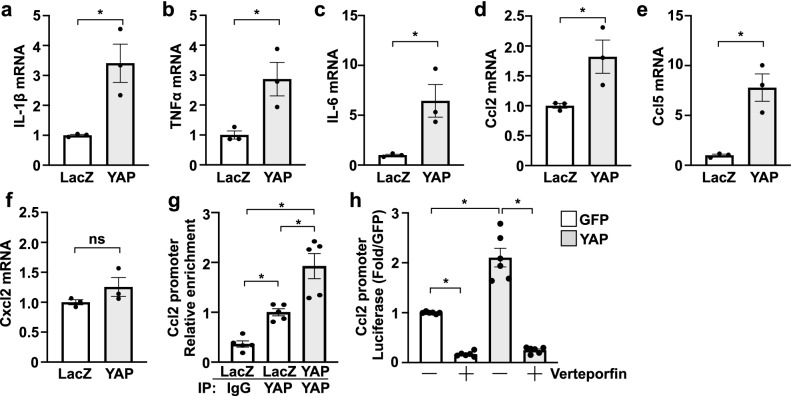
YAP promotes inflammatory-associated gene expression in cardiac fibroblasts. (**a–f**) Adenoviral-mediated expression of YAP in NRCFs increased select cytokine and chemokine mRNA expression. (**g**) ChIP analysis demonstrates YAP enrichment at the *Ccl2* gene promoter in NRCFs. (**h**) A Ccl2-luciferase reporter construct was used to detect promoter activation. Increased YAP expression significantly increased luciferase activity in HEK293 cells, which was significantly reduced in the presence of verteporfin (1 μM). N = 3 experimental replicates. *p < 0.05. *ns* not significant.

Our previous work demonstrated that endogenous fibroblast YAP is activated in the mouse heart following myocardial infarction^15^. To mimic this upregulation in vitro, we transduced neonatal rat cardiac fibroblasts with YAP(S127A) adenovirus or LacZ control (Fig. 4a,b). Our results demonstrate that increasing YAP expression causes the upregulation of fibrosis-associated gene expression (Fig. 4e–g). We also assessed whether endogenous YAP was required for the upregulation of fibrotic genes. We found that YAP depletion attenuated TGFβ1-induced upregulation of fibrotic genes (Fig. 4c,d,h–j). Because fibroblast expansion also contributes to myocardial fibrosis, we tested the effect of YAP on proliferation. Fibroblasts transduced with YAP had increased Ki67 positivity compared to control cells indicating that YAP activation promotes this response (Fig. 4k,l). We then questioned whether increased fibroblast YAP activation is sufficient to stimulate an inflammatory response, which is a hallmark of MI-induced pathology. Quantitative PCR revealed increased mRNA expression of the pro-inflammatory cytokines IL-1β, TNFα, and IL-6 in YAP transduced fibroblasts compared to control transduced cells (Fig. 5a–c). YAP expression also increased the expression of chemokines Ccl2 and Ccl5 in cardiac fibroblasts (Fig. 5d–f). The Ccl2 promoter contains multiple predicted TEAD binding motifs, which are present in human, mouse, and rat. To determine whether YAP binds to the promoter of Ccl2, we performed ChIP in cardiac fibroblasts using an anti-YAP antibody. We found that YAP-immunoprecipitated products were enriched with Ccl2 promoter DNA, which was increased further in samples with exogenous YAP expression (Fig. 5g). We next generated a Ccl2 reporter construct using a 1.5 kb sequence proximal to the first exon of the rat *Ccl2* gene. YAP(S127A) expression significantly increased luciferase activity in HEK293 cells compared to GFP, indicating transcriptional regulation of the Ccl2 promoter by YAP (Fig. 5h). Moreover, this response was abolished by treatment with the YAP inhibitor verteporfin^28^, indicating YAP-TEAD involvement in regulating Ccl2 gene expression (Fig. 5h). These data indicate that YAP activation in cardiac fibroblasts causes increased expression of inflammatory cytokines and chemokines that may promote inflammation in vivo.

### YAP transduced hearts had increased chemokine expression and macrophage abundance

**Figure 6:**
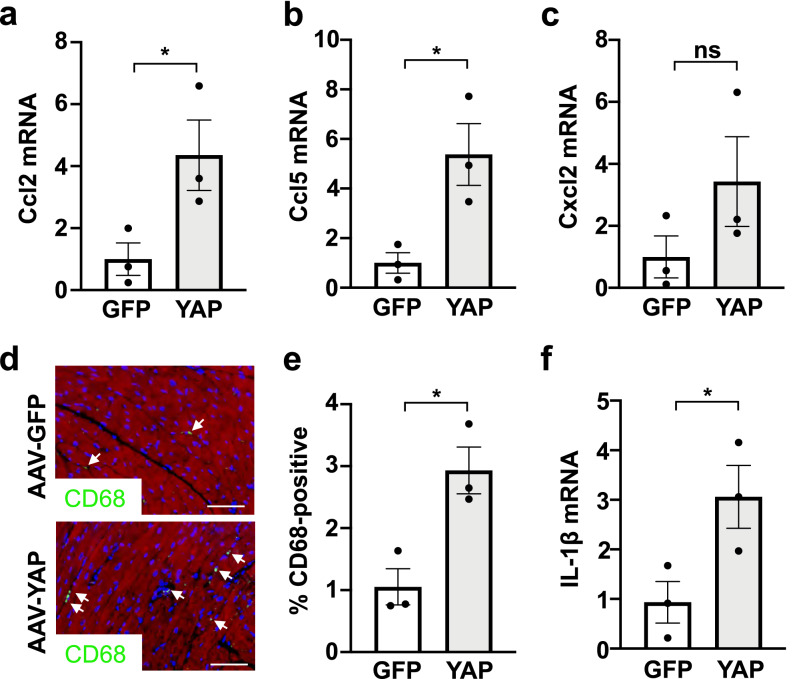
Transduction with AAV-hTCF21-FLAG-YAP upregulates inflammatory markers in the heart. Wild type C57BL/6J mice were administered AAV-hTCF21-GFP or AAV-hTCF21-FLAG-YAP. (**a–c**) RNA was isolated from LV tissue for qPCR analysis. (**d**,**e**) Hearts were sectioned for immunostaining to detect CD68 + macrophages (green), cardiac troponin T (red), and nuclei (DAPI). White arrows identify CD68-positive cells. Quantification of CD68-positive cells in panel (**e**). Scale bar, 100 μm. (**f**) IL-1b mRNA was measured in LV tissue by qPCR. N = 3 mice/group. *p < 0.05. *ns* not significant.

We next examined markers of inflammation in the AAV-hTCF21-GFP and AAV-hTCF21-FLAG-YAP transduced mice. Consistent with our cultured fibroblast results, YAP transduced hearts had higher levels of Ccl2 and Ccl5 mRNA compared to GFP control (Fig. 6a–c). In addition, we observed an increased presence of CD68-positive macrophages in myocardium from AAV-hTCF21-FLAG-YAP transduced mice compared to AAV-hTCF21-GFP controls (Fig. 6d,e). Expression of the pro-inflammatory cytokine IL-1β was also increased in YAP transduced hearts compared to GFP control hearts (Fig. 6f). Taken together, these results suggest that cardiac fibroblast YAP activation stimulates Ccl2 chemokine expression and promotes immune cell recruitment, which may enhance cardiac inflammation in vivo.

## Discussion

Fibroblasts direct ECM production and maintenance and play a critical role in orchestrating physiological wound healing in response to tissue injury^29^. Prolonged activation of fibroblasts (i.e. myofibroblast differentiation) can mediate pathological outcomes that ultimately impair heart contraction/relaxation and decrease cardiac output. Therefore, it is imperative to better understand the molecular mechanisms regulating myofibroblast differentiation and excessive fibrosis in order to improve heart function following stress and prevent the progression to failure.

Our recent work demonstrated that endogenous YAP is activated in cardiac fibroblasts in response to MI or neurohormonal stimulation^15^. Genetic disruption of YAP in cardiac fibroblasts resulted in attenuation of fibrosis and dysfunction following MI, which was associated with decreased expression of the transcriptional co-factor MRTF-A and ECM-related genes. Indeed, cell-based experiments showed that YAP depletion prevents AngII-induced myofibroblast differentiation, pro-fibrotic gene expression, and contractile properties that define myofibroblasts^2^. In addition, we showed that YAP regulates the proliferation of cardiac fibroblasts in response to MI, which likely contributes to the fibrotic response in vivo. In the current study, we demonstrate that in vivo fibroblast YAP activation increases markers of myofibroblast differentiation and collagen deposition. These results are consistent with previous work demonstrating that Lats1/2 deletion in cardiac fibroblasts initiates unrestrained fibrosis that is mediated by YAP/TAZ^30^, as well as a recent study demonstrating a p38-YAP-TEAD signaling complex responds to post-infarct ECM mimetics and mediates myofibroblast differentiation of cardiac fibroblasts^12^. Together these findings indicate a critical role for Hippo-YAP in modulating myofibroblast differentiation and cardiac fibrosis in relevant pre-clinical disease models.

In addition to providing structural support, fibroblasts also participate in cell-to-cell communication through the secretion of soluble factors including cytokines and chemokines, and therefore modulate inflammatory responses following tissue injury^14^. In response to MI, for example, the initial pro-inflammatory response is critical for wound healing and prevention of cardiac rupture. However, the resolution of inflammation and transition to a wound healing/pro-angiogenic phase is also required for proper repair. Prolonged and/or excessive inflammation, even at a low grade, can have detrimental effects and worsen remodeling and heart function. Communication between neutrophils, macrophages/monocytes, and cardiac fibroblasts plays an important role in the inflammatory response as fibroblasts are able to regulate the recruitment and activation status of circulating immune cells. Fibroblasts, among other resident heart cells, can secrete cytokines (e.g. IL-1β, TNFα) and chemokines (e.g. Ccl2, Ccl5) to promote inflammatory signaling^31^. Importantly, our results demonstrate that enhanced fibroblast YAP expression causes increased expression of IL-1β, TNFα, and IL-6 mRNA. Additionally, YAP upregulated expression of Ccl2 and Ccl5 in cultured cardiac fibroblasts as well as in vivo. This was associated with enhanced presence of macrophages and IL-1β expression in YAP transduced hearts. These data indicate that fibroblast YAP activation is sufficient to induce expression of established pro-inflammatory cytokines and chemokines and may serve to stimulate the recruitment of macrophages and other immune cells to the myocardium.

Ccl2 (MCP-1) expression is increased following MI and is a critical signal that promotes the recruitment of pro-inflammatory monocytes to the injured heart and stimulates pro-fibrotic effects^14,32,33^. Previous work has demonstrated that cardiac fibroblasts participate in leukocyte recruitment and are a significant source of Ccl2 post-MI^34^. Blockade of CCR2, the receptor for Ccl2, either in monocytes or systemically, has been shown to reduce inflammation and pathological cardiac remodeling after infarction, underscoring the importance of this chemokine signal in ischemic heart injury^35,36^. Our current findings using ChIP, qPCR, and luciferase reporter assays indicate that YAP occupies the Ccl2 promoter and exerts positive regulation of Ccl2 transcription in cardiac fibroblasts. This is not without precedent, as prior work has demonstrated that YAP occupies the *Ccl2* gene and increases Ccl2 expression in mouse liver^37^, mouse macrophages^38^, and a human hepatocyte cell line^39^. Induction of pro-inflammatory chemokines, including Ccl2, was also reported in a mouse model of fibroblast Lats1/2 deficiency^30^. Our data support this cardiac fibroblast YAP-mediated mechanism, which may modulate immune cell behavior in the heart. These findings add to a growing literature describing a complex and likely cell-type and context-dependent Hippo-YAP involvement in inflammatory regulation^38,40–47^.

We now demonstrate the feasibility of targeting cardiac fibroblast selective gene expression in vivo by leveraging AAV in combination with the hTCF21 promoter. To our knowledge, ours is the first study to augment the expression of YAP in cardiac fibroblasts in vivo. We acknowledge, however, that low-level exogenous YAP mRNA was detected in liver tissue, and off-target expression in other organs may occur using this AAV approach, which is a limitation of the study. Increased expression of active YAP was sufficient to increase the expression of cytokines and chemokines that are recognized drivers of inflammation in the heart. Additionally, YAP expression increased the presence of macrophages, as well as the extent of fibrosis in the heart, in the absence of additional stress. This was associated with a decline in cardiac function. Our recent study demonstrated that genetic inhibition of cardiac fibroblast YAP attenuated fibrosis and dysfunction post-MI, and our new data indicate that YAP occupies the *Ccl2* gene to promote Ccl2 expression. We hypothesize that the pro-fibrotic and pro-inflammatory functions of YAP in cardiac fibroblasts make it a potential therapeutic target to mitigate pathological remodeling after ischemic heart injury.

## Supplementary Information

Supplementary Information.
