# Supplementary material for: AAV-mediated YAP expression in cardiac fibroblasts promotes inflammation and increases fibrosis
Source: Sci Rep. 2021 May 18;11:10553. doi: 10.1038/s41598-021-89989-5 (PMC8131354; doi:10.1038/s41598-021-89989-5)

**AAV-mediated YAP expression in cardiac fibroblasts promotes inflammation and increases fibrosis**

Jamie Francisco, Yu Zhang, Yasuki Nakada, Jae Im Jeong, Chun-Yang Huang, Andreas Ivessa, Shinichi Oka, Gopal J. Babu, and Dominic P. Del Re

Department of Cell Biology and Molecular Medicine, Cardiovascular Research Institute, Rutgers New Jersey Medical School, Newark, NJ

**<u>Supplementary Materials:</u>**

**Supplementary Table S1**

**Supplementary Table S2**

**Supplementary Table S3**

**Supplementary Figure S1**

**Supplementary Figure S2**

**Supplementary Figure S3**

**Table S1. Echocardiographic analysis of mice 4 weeks after AAV administration.**

| Parameter | AAV-hTCF21-GFP | AAV-hTCF21-FLAG-YAP(S127A) |
|---|---|---|
| *n* | 4 | 4 |
| LVIDd (mm) | 3.64±0.10 | 3.58±0.18 |
| LVIDs (mm) | 2.15±0.05 | 2.55±0.15* |
| IVSd (mm) | 1.02±0.05 | 0.95±0.05 |
| PWTd (mm) | 0.99±0.04 | 0.95±0.02 |
| LVEF(%) | 79.4±0.7 | 63.6±3.3* |

Data are presented as mean ± S.E.M. * $p<0.05$ versus AAV-hTCF21-GFP. LVIDd (left ventricular end diastolic dimension), LVIDs (left ventricular end systolic dimension), IVSd (diastolic septal wall thickness), PWTd (diastolic posterior wall thickness), LVEF(%) (left ventricular ejection fraction).

**Table S2. Echocardiographic analysis of mice 8 weeks after AAV administration.**

| Parameter | AAV-hTCF21-GFP | AAV-hTCF21-FLAG-YAP(S127A) |
| --- | --- | --- |
| *n* | 4 | 4 |
| LVIDd (mm) | 3.76±0.17 | 4.05±0.11 |
| LVIDs (mm) | 2.46±0.17 | 3.14±0.10* |
| IVSd (mm) | 1.00±0.02 | 0.89±0.07 |
| PWTd (mm) | 0.89±0.05 | 0.84±0.02 |
| LVEF(%) | 72.1±2.2 | 53.3±1.9* |

Data are presented as mean ± S.E.M. * $p<0.05$ versus AAV-hTCF21-GFP. LVIDd (left ventricular end diastolic dimension), LVIDs (left ventricular end systolic dimension), IVSd (diastolic septal wall thickness), PWTd (diastolic posterior wall thickness), LVEF(%) (left ventricular ejection fraction).

**Table S3. Echocardiographic analysis of mice 12 weeks after AAV administration.**

| Parameter | AAV-hTCF21-GFP | AAV-hTCF21-FLAG-YAP(S127A) |
|---|---|---|
| *n* | 4 | 4 |
| LVIDd (mm) | 3.93±0.11 | 3.88±0.03 |
| LVIDs (mm) | 2.70±0.13 | 3.00±0.08 |
| IVSd (mm) | 0.89±0.03 | 0.92±0.08 |
| PWTd (mm) | 0.83±0.08 | 0.85±0.06 |
| LVEF(%) | 67.8±2.3 | 54.0±2.8* |

Data are presented as mean ± S.E.M. * $p<0.05$ versus AAV-hTCF21-GFP. LVIDd (left ventricular end diastolic dimension), LVIDs (left ventricular end systolic dimension), IVSd (diastolic septal wall thickness), PWTd (diastolic posterior wall thickness), LVEF(%) (left ventricular ejection fraction).

**Supplementary Figure S1. Postmortem analysis of AAV-transduced mice.** Wild-type C57BL/6J mice were administered AAV-hTCF21-GFP or AAV-hTCF21-FLAG-YAP(S127A). (**a-d**) Immediately following sacrifice, heart weight, left ventricle weight, lung weight, and liver weight were measured and normalized to tibia length. (**e**) Hearts were fixed, sectioned, and stained with H&E. Representative images using 1x and 20x magnifications are shown. Scale bars, 1 mm and 200 μm, respectively. N = 4 mice/group. ns = not significant.

**Supplementary Figure S2. Characterization of livers from AAV-transduced mice.** Wild-type C57BL/6J mice were administered AAV-hTCF21-GFP or AAV-hTCF21-FLAG-YAP(S127A). (**a** and **b**) RNA was isolated from heart and liver tissue and qPCR performed to determine relative amounts of GFP and FLAG-Yap. (**c**) Endogenous Yap mRNA was determined in liver tissue. (**d** and **e**) Total YAP protein was determined by western blot using liver extracts. (**f** and **g**) Livers were stained with picrosirius red to visualize collagen deposition. (**f**) The percent of positive staining was determined. Representative images are shown in panel **g**. Scale bar, 200 μm. (**h-j**) Fibrosis-related gene expression in liver tissue was analyzed by qPCR. N = 3 mice/group. *, $p<0.05$. ns = not significant. Full-length blots are presented in Supplementary Figure S3.

**Supplementary Figure S3. Full-length western blot images.**

Supplementary Figure S1.

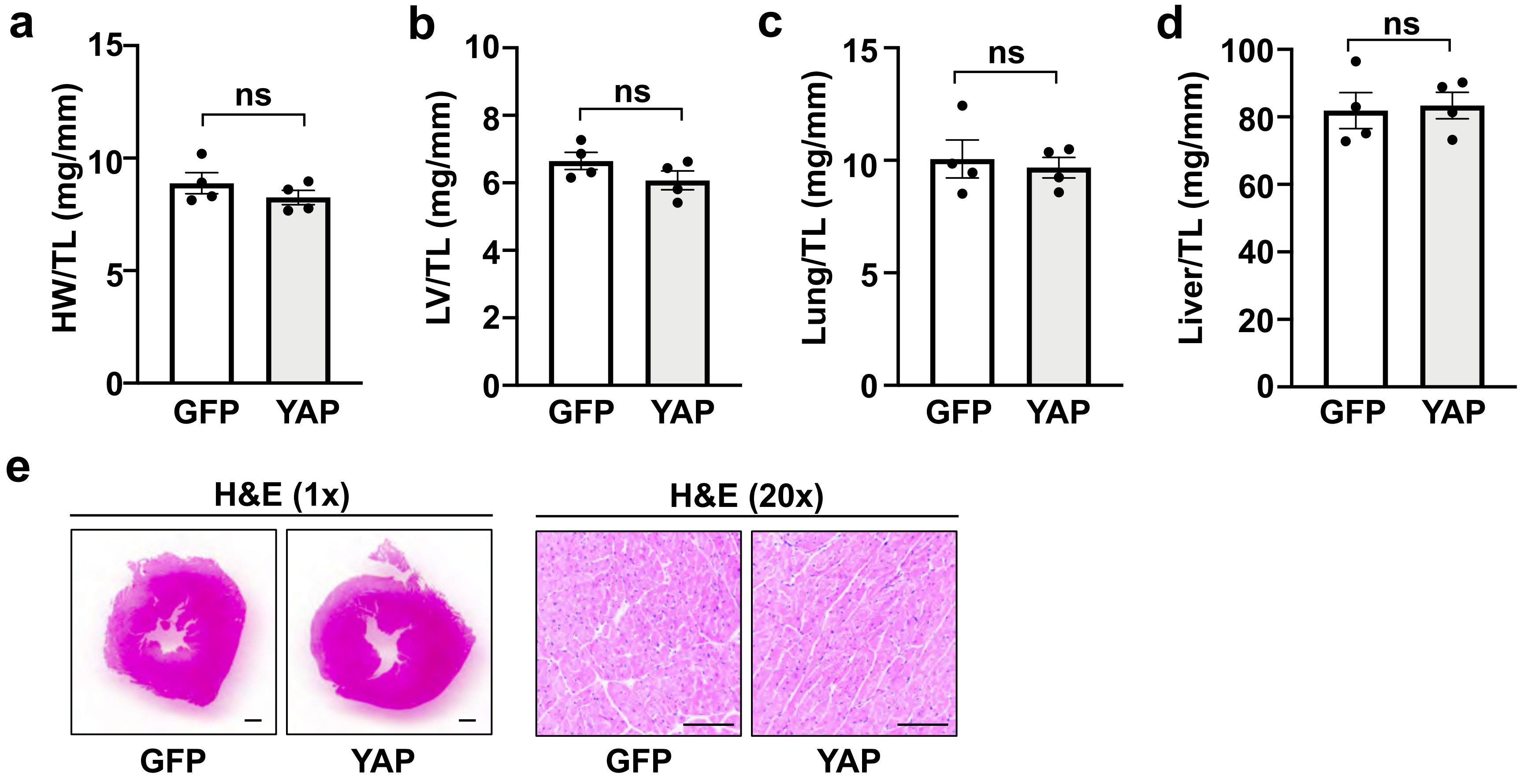

Supplementary Figure S2.

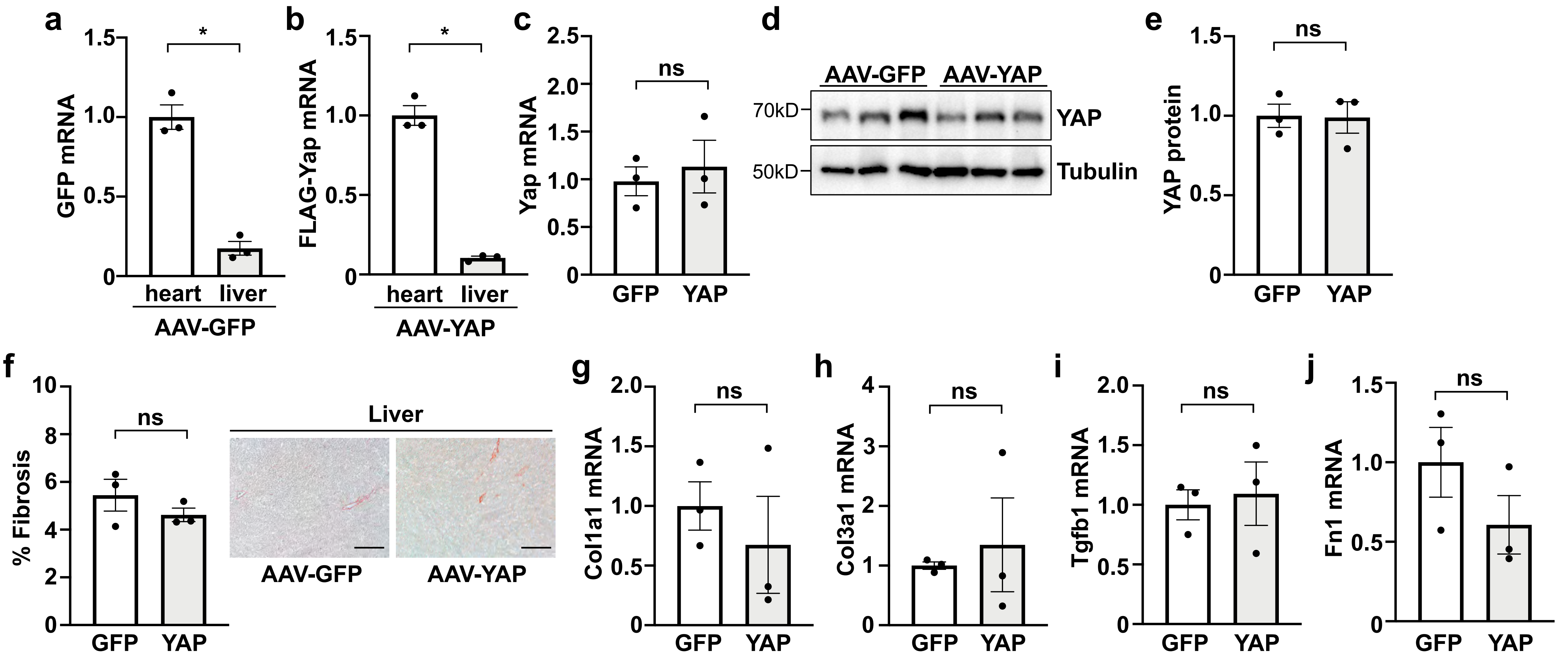

**Supplementary Figure S3.**

**Figure 1a**

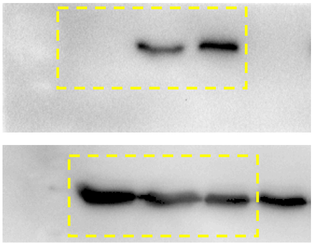

**Figure 1b**

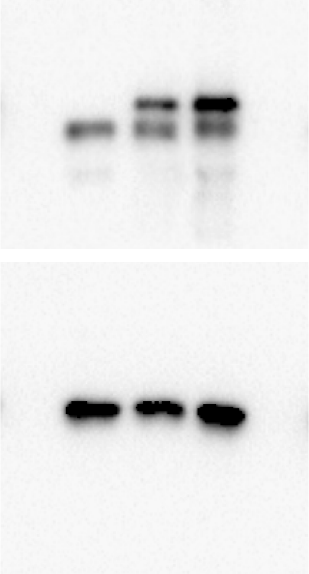

**Figure 2e**

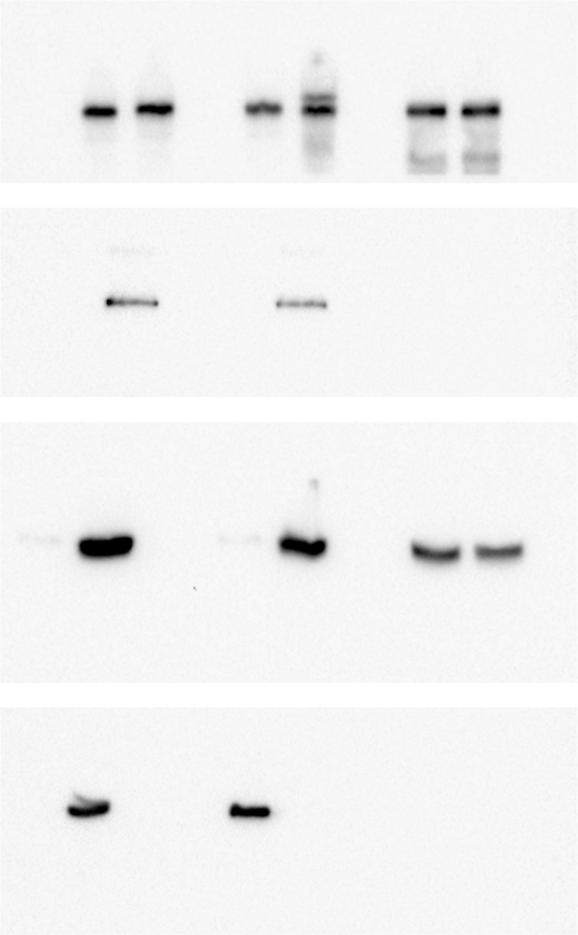

**Figure 4a**

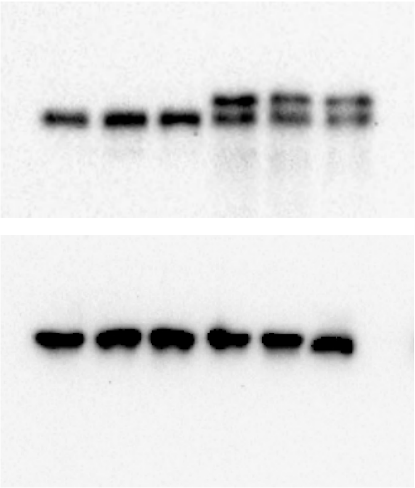

**Figure 4c**

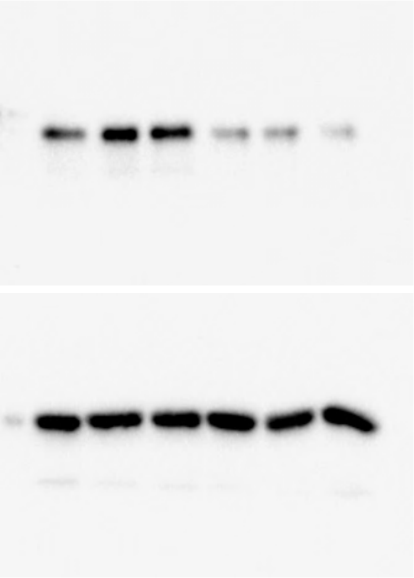

**Figure S2.**

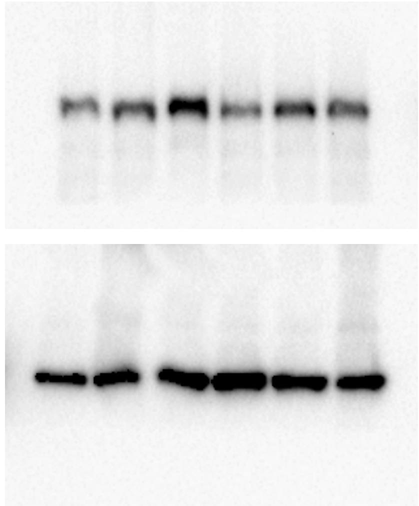

Supplement: Supplementary file 1 — Supplementary Information. [file 41598_2021_89989_MOESM1_ESM.pdf]
